# North Staffordshire Infirmary

**Published:** 1914-02-07

**Authors:** A. H. Heath

**Affiliations:** Kidsgrove, Stoke-on-Trent


### THE EDITOR'S LETTER-BOX.

### The North Staffordshire Infirmary.

### Letter from its late President.

*To the Editor of* The Hospital.

Sir,—In your reference to the annual meeting of this hospital in your last week's issue you quote me, in supporting the annual report, as saying "that the accommodation for operations was worse than that of any first-class hospital in the United Kingdom." Lest these words should convey an altogether wrong impression, which would have been made clear if my speech had been further reported, I am anxious to explain that our existing operating theatre lacks nothing in itself, and cannot be improved for operator or patient. We only require *more* accommodation, and this is to be provided at once.

During the last three years no less a sum than £35,000 has been subscribed, and nearly all spent in putting the infirmary in thoroughly good order; and the second operating theatre would have been provided as part of the scheme if it had been possible to decide on the site it was to occupy. This has now been agreed to, and the matter will be attended to at once. Our hospital is now in every department quite up to date and, we are pleased to think, will satisfy the most exacting expert. A far keener interest is taken in the work done there and a wider sympathy aroused in the district than ever before.

During the current year a great effort is to be made to put it on a sound financial basis, as since the demand on the accommodation has greatly increased the present revenue falls short of the requirements.—Yours, etc.,

A. H. Heath.

Kidsgrove, Stoke-on-Trent.

January 31, 1914.

[We have pleasure in publishing Colonel Heath's letter of explanation, and we give publicity to the testimony it bears to the encouraging results of the splendid efforts Colonel Heath made during the time he was President of this hospital. Our quotation was taken from the report of a contemporary, and we are glad to have Colonel Heath's assurance that as it stood it required material modification in the direction indicated by his letter. We have every confidence that the additional accommodation for operations will be speedily forthcoming, and that the awakening of public opinion under Colonel Heath and his successors to the importance of this hospital will ensure its maintenance at a high standard of efficiency.—Ed., The Hospital.]

